# Pharmacokinetics of Belimumab in Children With Systemic Lupus Erythematosus

**DOI:** 10.1002/cpdd.889

**Published:** 2020-11-27

**Authors:** Richard Dimelow, Beulah Ji, Herbert Struemper

**Affiliations:** ^1^ GSK Clinical Pharmacology Modeling & Simulation Stevenage Hertfordshire UK; ^2^ GSK Clinical Pharmacology Modeling & Simulation Research Triangle Park North Carolina USA

**Keywords:** modeling and simulation, pediatrics (PED), pharmacokinetics and drug metabolism, population pharmacokinetics, rheumatology

## Abstract

The phase 2 placebo‐controlled, double‐blind PLUTO trial characterized the pharmacokinetics of belimumab plus standard systemic lupus erythematosus (SLE) therapy in patients with childhood‐onset SLE (cSLE) and demonstrated similar efficacy and safety to that in adult SLE. Patients with active cSLE aged 5‐17 years were randomized to intravenous belimumab 10 mg/kg every 4 weeks (n = 53). A linear 2‐compartment population pharmacokinetics (popPK) model with first‐order elimination was developed, and an exploratory exposure‐response analysis assessed the impact of between‐patient exposure variability on clinical response (SLE Responder Index 4 [SRI4]) in week 52, and occurrence of serious adverse events during the study. The popPK model estimated clearance of 158 mL/day, steady‐state volume of distribution of 3.5 L, terminal half‐life of 16.3 days, and distribution half‐life of 0.8 days in the overall population. Fat‐free mass (FFM) better characterized the pharmacokinetics than total body weight and was more consistent with allometric scaling theory; belimumab pharmacokinetics were largely determined by FFM. Age, sex, disease activity, and concomitant medication had no impact on pediatric belimumab exposure after accounting for body size. Individual and median steady‐state pediatric pharmacokinetic profiles were similar to known adult profiles and pediatric exposure estimates for belimumab 10 mg/kg intravenously were consistent with adult exposures. Exposures were similar between SRI4 responders and nonresponders, and patients who did or did not experience a serious adverse event. There was no clinically relevant correlation between exposure and efficacy or safety, confirming belimumab 10 mg/kg intravenous dose every 4 weeks as appropriate for pediatric patients with cSLE.

Systemic lupus erythematosus (SLE) is a chronic inflammatory autoimmune disease that commonly manifests with a variety of symptoms and laboratory abnormalities.^1^, ^2^ Childhood‐onset SLE (cSLE) is rare, with a reported prevalence of between 3.3 and 24 per 100 000 children^1^, ^3^ and typically has a more severe clinical course than that observed in adults.^1^

The heterogenous and complex nature of SLE means that diagnosis is often delayed until progression is advanced and several organs have become affected.^4^ This is particularly pronounced in cSLE, in which children frequently experience higher disease activity and a faster rate of SLE damage accrual than adults.^5^, ^6^ Some clinical manifestations also occur more frequently in children; for example, the frequency of hematological abnormalities and mucosal ulcers is approximately twice as high in children as in adults.^6^

Patients with SLE have an elevated plasma concentration of the B Lymphocyte Stimulator (BLyS), with higher BLyS levels known to positively correlate with SLE disease activity.^7^ Belimumab is a recombinant immunoglobulin G (IgG) 1λ human monoclonal antibody that targets BLyS and is approved for adult SLE as 10 mg/kg intravenously every 28 days and 200 mg/week subcutaneous formulations and in pediatric patients with cSLE as a 10 mg/kg intravenous formulation.^8^, ^9^, ^10^, ^11^

The population pharmacokinetics (popPK) of belimumab in adult patients with SLE following intravenous and subcutaneous administration has been previously characterized using a 2‐compartment model with first‐order absorption, distribution, and elimination.^12^, ^13^ Body weight and body mass index (BMI) were the most PK‐relevant covariates of belimumab clearance (CL) following intravenous and subcutaneous administration.^12^, ^13^ At exposures following 10 mg/kg intravenously every 28 days or 200 mg subcutaneously weekly, belimumab was predicted to be in excess of circulating levels of BLyS,^12^ and the pharmacological and efficacy response was observed to be close to saturation.^13^

The efficacy and safety of belimumab in pediatric patients with cSLE have recently been demonstrated in the PLUTO study,^14^ resulting in belimumab becoming the first approved treatment for SLE in this patient population. The PLUTO study consisted of age‐staggered PK cohorts, which provided denser PK sampling followed by a single efficacy cohort. The double‐blind, randomized, placebo‐controlled PK cohorts were an important design element, in that the data from these 2 cohorts could be incorporated into the overall efficacy analysis, which would not have been possible with traditional open‐label PK cohorts; this was an important feature considering an enrollment rate of approximately 21 participants per year in this indication. The published results^14^ provide a descriptive summary of the observed pre‐ and postdose belimumab concentrations. Here, we report the popPK analysis from the 52‐week treatment period of the PLUTO study, which further characterizes the size and variability of belimumab clearance and distribution in pediatric patients through a model‐based compartmental analysis. This analysis enables a more detailed assessment of belimumab exposure at steady state, including the minimum, maximum, and average belimumab concentrations over a 28‐day dosing period. In addition, the allometric dependence of body size on belimumab clearance is explored, comparing body weight with fat‐free mass, which may be the more appropriate covariate for monoclonal antibodies that may not readily distribute into adipose tissue. The exposure‐response relationship for efficacy and safety assessed in this pediatric patient population is also assessed.

## Methods

### Ethics Approval

The study protocols and amendments were reviewed and approved by a national, regional, or investigational center ethics committee or institutional review board, in accordance with the International Council for Harmonisation of Technical Requirements for Registration of Pharmaceuticals for Human Use Good Clinical Practice and applicable country‐specific and patient privacy requirements. The study was conducted in accordance with the ethical principles outlined in the Declaration of Helsinki 2008. Written informed consent was obtained from each patient's parent(s) or legal guardian prior to randomization, and written assent, in age‐appropriate language, was obtained from each study patient in accordance with local regulations.

### Objectives

The objectives of this analysis were to develop a popPK model to characterize the PK disposition of belimumab following intravenous administration in pediatric patients with cSLE, evaluate the effect of selected covariates on key PK parameters, explore the exposure‐response relationships with respect to efficacy and safety, and compare belimumab exposure in pediatric and adult patients with SLE.

### Study Design

The design of this phase 2 multicenter, randomized, double‐blind, placebo‐controlled study (PLUTO; ClinicalTrials.gov identifier NCT01649765; GSK study BEL114055) has been previously described.^15^ Briefly, eligible patients aged 5‐17 years with active cSLE were randomized to belimumab 10 mg/kg intravenously or placebo, plus standard SLE therapy administered on days 0, 14, and 28, then every 28 days until week 48, followed by a final evaluation in week 52. The primary efficacy end point was the SLE Responder Index 4 (SRI4) response rate in week 52. Patients were enrolled into 1 of 3 cohorts: cohort 1 for patients aged 12‐17 years, cohort 2 for patients aged 5‐11 years, and cohort 3 open to both age groups, although only patients aged 12‐17 years were enrolled in this cohort. PK samples were collected on the day of dosing on days 0, 14, 28, 56, 168, and 364, with additional PK samples collected following the first 2 doses for cohorts 1 and 2 to enable a more detailed characterization of the distribution and elimination of belimumab in pediatric patients. The names and locations of all sites that participated in this study are provided in Table S1.

### Assay Methods

Serum samples were analyzed by Covance, Inc. (Princeton, New Jersey) using a validated method based on sample dilution followed by electrochemiluminescence immunoassay. The assay has been described in detail previously^12^ and follows the recommendations and best practices for ligand‐binding assays.^16^, ^17^ A 96‐well plate coated with biotinylated BLyS and preblocked for nonspecific binding sites captured belimumab in the assay sample. Belimumab was detected by the sequential addition of a proprietary rabbit antibelimumab primary antibody followed by a SULFO‐TAG‐conjugated goat antirabbit secondary antibody (Meso Scale Discovery, Gaithersburg, Maryland). The bound SULFO‐TAG moiety produces the luminescence signal, which establishes the belimumab concentration calculated by a nonlinear regression curve established with belimumab reference standard. The calibrator standards for belimumab concentrations ranged from 50 to 16 000 ng/mL, and at each calibrator concentration the percent bias of nominal values and the coefficient of variation for repeat values were within 20% (25% at the lower and upper limits of quantification), demonstrating a suitable level of accuracy. Using this assay, the lower limit of quantification (LLQ) for belimumab was 0.1 µg/mL.

### Modeling

#### Objectives

A popPK model was fitted to the pediatric study results to characterize the PK and exposure‐response relationships in a pediatric cSLE population. Key PK parameters included the systemic CL, volume of distribution at steady state (V_ss_), volume of distribution during the terminal phase (V_z_), distribution‐phase half‐life (T_1/2α_), and terminal phase half‐life (T_1/2β_) of belimumab. Exposure was described by the maximum concentration at steady state (C_max_), minimum concentration at steady state (C_min_), average concentration at steady state (C_avg_), and area under the concentration‐time curve over a 28‐day dosing interval at steady state (AUC) after belimumab dosing.

### Data Analysis

Data manipulation, model development, and analysis were conducted within the GSK Modeling and Analysis Platform, incorporating NONMEM version 7.3 (ICON, Dublin, Ireland), PsN version 4.6.0 (Uppsala University, Uppsala, Sweden), and R version 3.2.5 (free open‐source software). The first‐order conditional estimation method with interaction in NONMEM version 7.3 was used to estimate the parameters of the popPK model through maximizing the likelihood function (equivalent to minimizing the objective function).

### PopPK Model Development

The popPK model was based on that developed for an adult SLE population^12^ and was a 2‐compartment structural model with constant‐rate infusion and first‐order distribution and elimination. The unmodified starting model was initially used to evaluate pediatric PK using population parameters fixed at their adult‐derived estimates (MAXEVAL = 0 argument in NONMEM). To correct for a small underprediction of the pediatric data, the model was then adjusted for pediatric covariate distributions, and the population parameters were re‐estimated for the pediatric population. The effect of body size on CL and volume of distribution was further investigated post hoc. The full model was reduced by identifying the covariates in the model that were not PK‐relevant for pediatric patients using a variation of the full covariate model approach^18^ as follows. The ratio of the model parameter at the 10th and 90th percentiles of the covariate, relative to the model parameter at the median covariate value (ratios R_10_ and R_90_, respectively), was chosen as a quantifiable measure of the PK relevance of the covariate effect accounting for the width of the distribution of the underlying covariate values. If either R_10_ or R_90_ was outside the range of 0.8‐1.25, then the covariate was considered PK‐relevant and retained in the model. If both R_10_ and R_90_ and their 95% confidence intervals (CIs) were within the range of 0.8‐1.25, then the covariate was not PK‐relevant and was removed from the model. If at least 1 of the ratio 95%CIs extended outside the range of 0.8‐1.25, the decision to retain the covariate was based on the covariate parameter uncertainty: if the parameter 95%CI was wholly positive or negative, then the covariate was considered PK‐relevant and retained; otherwise the covariate was removed. Additional covariates with the potential to correlate with exposure, including demographic information (age, race, Hispanic ethnicity), SLE disease activity (baseline levels for BLyS, C‐reactive protein, anti‐double stranded DNA antibodies complement C3 and C4, naive B cells, Safety of Estrogen in Lupus Erythematosus National Assessment–SLE Disease Activity Index [SELENA‐SLEDAI] score), liver function measures at baseline, and use of concomitant mediations were then tested for a significant impact on the CL parameter of the reduced model. The likelihood ratio test was used to determine whether a reduction in the objective function was sufficiently large to justify the inclusion of these additional covariates. For the addition of a single degree of freedom, this reduction was required to be at least 3.84 objective function points at the 95% level of certainty.

### Model Testing, Validation, and Selection

Empirical Bayes estimates for individual parameter values and corresponding PK predictions were used to assess the model fit and to identify any model misspecification. A visual predictive check was performed on the final model, in which the data set was simulated 1000 times and compared with the observed PK values to check for consistency. The final model parameter estimates with associated precision were confirmed using a bootstrap method: the final model was refitted to 2000 pseudo‐data sets, each data set having sampled n = 53 patients with replacement from the actual study data set, so that each data set contained the same number of patients as the original. The model was successfully fitted to 1694 of the sampled data sets and the distribution of the fitted parameter values used to characterize the precision around the average point estimate.

### Steady‐State PK and Exposure‐Response Analysis

The individual PK parameter estimates were used to predict the steady‐state concentration‐time profiles for each patient in the study who received belimumab 10 mg/kg every 28 days and were characterized by the steady‐state C_min_, C_avg_, and C_max_. The relationship between individual steady‐state concentrations and individual‐level efficacy and safety end points was explored: the SRI4 and the occurrence of a serious adverse event (SAE) by week 52.

## Results

### Patient Disposition

A total of 93 patients (94.6% female; median age, 15.0 years [range, 6‐17 years]) were randomized to treatment with belimumab (n = 53) or placebo (n = 40). Of those randomized to belimumab, 10 patients were in the 5‐ to 11‐year age group (cohort 2), and 43 patients were in the 12‐ to 17‐year age group (cohorts 1 and 3), and an overview of their demographics and baseline characteristics is shown in Table 1. After excluding all observed concentrations that were below the limit of quantification or not analyzed, a total of 560 PK observations were recorded from the 53 patients who received belimumab in the double‐blind 52‐week treatment period.

**Table 1:** Demographics and Baseline Characteristics of Patients Receiving Belimumab (n = 53)

Characteristic	Baseline Age 5‐11 Years (Cohort 2), n = 10	Baseline Age 12‐17 Years (Cohorts 1 and 3), n = 43	Overall, n = 53
Age, y			
Mean (SD)	9.5 (1.7)	14.5 (1.7)	13.6 (2.6)
Median (min‐max)	10 (6‐11)	15 (12‐18^a^)	14 (6‐18)
Weight, kg			
Mean (SD)	32.0 (10.5)	56.8 (14.5)	52.1 (16.9)
Median (min‐max)	29.8 (17.0‐55.2)	53.2 (31.5‐85.5)	52.3 (17.0‐85.5)
Female, n (%)	10 (100)	39 (91)	49 (92)
BMI, kg/m^2^			
Mean (SD)	18.4 (2.7)	22.8 (4.4)	22.0 (4.5)
Median (min‐max)	17.8 (15.4‐23.9)	22.1 (16.2‐34.0)	21.4 (15.4‐34.0)
FFM, kg			
Mean (SD)	22.1 (6.2)	37.2 (7.9)	34.3 (9.6)
Median (min‐max)	20.6 (12.6‐35.0)	35.6 (22.6‐57.2)	34.4 (12.6‐57.2)
BlyS, ng/mL			
Mean (SD)	1.3 (1.0)	0.89 (0.85)	0.98 (0.89)
Median (min‐max)	0.95 (0.48‐3.84)	0.66 (0.16‐4.31)	0.70 (0.16‐4.31)
White blood cell count, 10^9^/L			
Mean (SD)	6.7 (2.5)	6.2 (2.6)	6.3 (2.5)
Median (min‐max)	6.7 (2.4‐10.6)	5.8 (2.5‐13.0)	5.9 (2.4‐13.0)
Proteinuria, n (%)			
<0.5 mg/mg	10 (100)	39 (91)	49 (92)
≥0.5 mg/mg	0 (0)	4 (9)	4 (8)
IgG, g/L			
Mean (SD)	16.7 (5.2)	15.4 (6.3)	15.6 (6.1)
Median (min‐max)	16.2 (10.7‐24.8)	14.0 (4.1‐31.2)	14.5 (4.1‐31.2)
eGFR, mL/min/1.73 m^2^			
Mean (SD)	118 (18)	108 (21)	110 (21)
Median (min‐max)	124 (94‐141)	103 (74‐155)	105 (74‐155)

BlyS, B Lymphocyte Stimulator; BMI, body mass index; eGFR, estimated glomerular filtration rate; FFM, fat‐free mass; IgG, immunoglobulin G.

^a^ One patient was aged 17 years at the time of screening.

### Final Model

**Figure 1:**
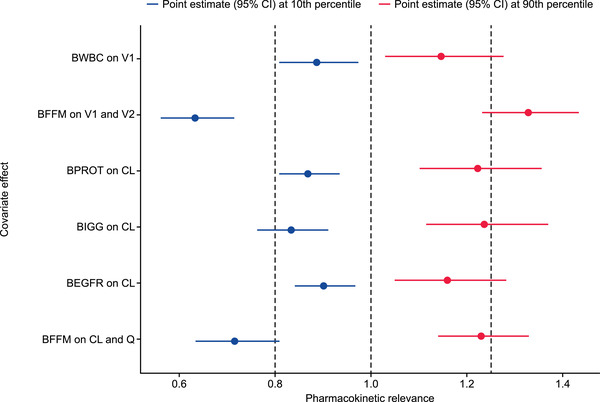
Covariate parameter estimates for the final popPK model. BEGFR, baseline estimated glomerular filtration rate; BFFM, baseline fat‐free mass; BIGG, baseline serum immunoglobulin G; BPROT, baseline proteinuria; BWBC, baseline white blood cell count; CI, confidence interval; CL, clearance, popPK, population pharmacokinetics; Q, intercompartment clearance; V1, central volume of distribution; V2, peripheral volume of distribution. Point estimates for the size of the covariate effect are shown for the 10th (blue points) and 90th (red points) percentiles in the observed covariate values. The uncertainty in the point estimates due to the precision in the covariate parameter estimates is shown (horizontal bars).

**Figure 2:**
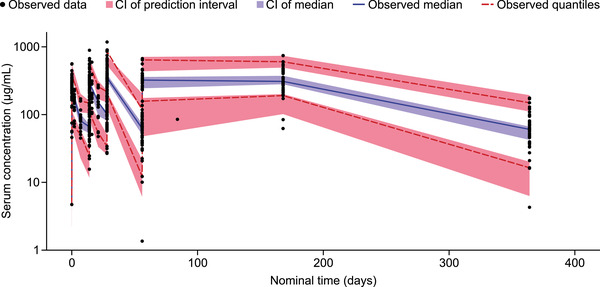
Visual predictive check of the final popPK model with respect to nominal time. CI, confidence interval; popPK, population pharmacokinetics. Serum concentrations below the limit of quantification were not included in the analysis.

The final linear 2‐compartment model, with first‐order distribution and elimination, had population estimates for CL of 158 mL/day, V_ss_ of 3549 mL calculated as the sum of the model compartment central (V1) and peripheral (V2) volumes of distribution, and T_1/2α_ and T_1/2β_ of 0.8 and 16.3 days, respectively. Baseline fat‐free mass (FFM), calculated as a function of body weight, BMI, and sex,^19^ most accurately described the effect of body size on CL, intercompartment flow rate (Q), and V1 and V2 volumes of distribution. Point estimates of the exponents for the CL and Q (0.691) and for V1 and V2 (0.944) were consistent with expected allometric values (Table 2).^20^ Age had no significant effect on CL after accounting for the effects of body size. Additional PK‐relevant covariates that were retained in the final model were the effect of baseline IgG, proteinuria, estimated glomerular filtration rate on CL, and the effect of baseline white blood cell count on V1 (Table 2). The clinical relevance of covariates included in the final model was confirmed with the full covariate method (Figure 1). Between‐patient variability on CL, V1, and V2 not explained by the covariate effects was modeled as being log‐normally distributed across the population. A proportional residual model with a small additive component was used to characterize the residuals. It was necessary to fix the additive component to the estimated value from the adult popPK model (variance, 0.0139 (ug/mL)^2^; standard deviation, 0.118 µg/mL), which is consistent with the LLQ of 0.1 µg/mL, because the lowest observed concentrations in the pediatric data set were generally ≥10 µg/mL (Figure 2), meaning that an additive component could not be estimated from the data. The contribution of several potentially relevant covariates (see Methods section for the full list) to the model was assessed to identify whether their inclusion resulted in an improved fit to the observed PK. These additional covariates, which included age, race, SLE disease activity, liver function measures, and use of concomitant medications, did not sufficiently contribute to the fit to the data, and so were not included in the final model.

**Table 2:** Parameter Estimates of the Final popPK Model

Parameter	Implementation	Model Point Estimate (%RSE,^a^ 95%CI)	Bootstrap Estimates,^b^ Median (95%CI)
Fixed effects			
CL (mL/day)		158 (3.6%, 147–169)	157 (142–169)
Effect of BFFM	× (BFFM/34.4)^θ^	0.691 (18.6%, 0.439–0.942)	0.691 (0.418–0.963)
Effect of BEGFR	× (BEGFR/105.21)^θ^	0.561 (34.4%, 0.183–0.939)	0.510 (0.079–0.966)
Effect of BIGG	× (BIGG/14.5)^θ^	0.396 (24.8%, 0.204–0.588)	0.415 (0.237–0.639)
Effect of BPROT	× (BPROT/0.132)^θ^	0.184 (26.4%, 0.089–0.279)	0.176 (0.082–0.289)
V1 (mL)		1927 (4.0%, 1777–2077)	1932 (1782–2090)
Effect of BFFM	x (BFFM/34.4)^θ^	0.944 (13.5%, 0.694–1.19)	0.941 (0.695–1.220)
Effect of BWBC	x (BWBC/5.9)^θ^	0.245 (39.9%, 0.054–0.437)	0.242 (0.020–0.426)
Q (mL/day)		701 (18.6%, 445–957)	700 (150–931)
Effect of BFFM	× (BFFM/34.4)^θ^	—	—
V2 (mL)	× (BFFM/34.4)^θ^	1622 (15.2%, 1138–2105)	1635 (1128–2157)
Effect of BFFM		—	—
Between‐patient variability (log‐scale variance and covariance parameters)			
ω^2^_CL_		0.0477 (28.8%, 0.0207–0.0746)	0.0407 (0.0175–0.0674)
ω^2^_V1_		0.0620 (24.7%, 0.0320–0.0920)	0.0572 (0.0306–0.0888)
ω^2^_CL/V1_		0.0366 (34.1%, 0.0122–0.0611)	0.0347 (0.0117–0.0590)
ω^2^_V2_		0.434 (29.1%, 0.187–0.681)	0.418 (0.203–1.413)
Residual variability (variance parameters)			
σ^2^_proportional_		0.0884 (18.3%, 0.0568–0.120)	0.0871 (0.0603–0.120)
σ^2^_additive_		0.0139 (fixed)	0.0139 (fixed)

%RSE, relative standard error as percentage of estimate; BEGFR, baseline estimated glomerular filtration rate; BFFM, baseline fat‐free mass; BIGG, baseline immunoglobulin G levels; BPROT, baseline proteinuria level; BWBC, baseline white blood cell count; CI, confidence interval; CL, clearance; Q, intercompartment clearance; V1, volume of distribution for the central compartment; V2, volume of distribution for the peripheral compartment.

^a^ Calculated as (standard error/mean) × 100%.

^b^ Bootstrap parameters based on 1694 successful minimizations from 2000 attempts.

### Final Model Validation

Individual and population model predictions matched the observed data, with the conditional weighted residuals uniform and unbiased with respect to time and individual predictions (Figure S1). A visual predictive check confirmed that variation in the predicted serum concentration in the final model was consistent with observed values (Figure 2).

### Steady‐State PK

**Figure 3:**
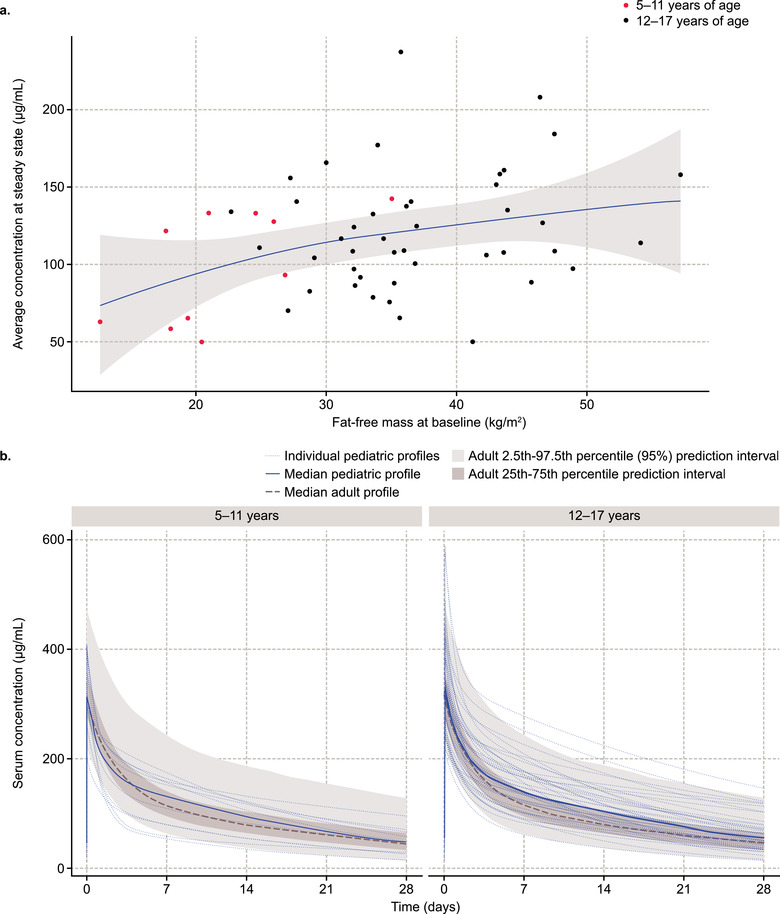
Belimumab exposure at steady state. (a) Steady‐state C_avg_ versus FFM at baseline by age group. (b) Simulated steady‐state PK profiles by age group compared with adult profiles. C_avg_, average serum drug concentration; FFM, fat‐free mass; PK, pharmacokinetics. Observed belimumab serum concentrations below the limit of quantification were not included in the analysis data set when calculating the individual pediatric profiles.

The PK parameters CL and V_ss_, were slightly lower for the patients aged 5‐11 years compared with those aged 12‐17 years consistent with allometry, and the T_1/2β_ values were similar between the age groups (Table 3). Total belimumab exposure at steady state over the 28‐day dosing period (AUC_ss_) was 21% lower in patients aged 5‐11 years compared with those aged 12‐17 years, in line with allometric principles for weight‐proportional dosing (10 mg/kg intravenously). PK parameters were generally consistent with those found in adults; however, the T_1/2α_ in adult patients was estimated to be more than double that of pediatric patients (Table 3). Belimumab exposure at steady state was positively correlated with patient FFM at baseline (Figure 3A), although large variability in the observed exposures was still apparent at any given body size. Simulated steady‐state PK profiles for all individual belimumab‐treated pediatric patients were consistent with adult profiles (Figure 3B); almost all pediatric profiles were completely contained in the adult 95% prediction interval, and median profiles were similar for adult and pediatric patients.

**Table 3:** Summary of Individual Belimumab PK and Exposure Parameters by Baseline Age Group

Parameter	Baseline Age 5‐11 Years (Cohort 2), n = 10	Baseline Age 12‐17 Years (Cohorts 1 and 3), n = 43	Total Pediatric Population, n = 53	Adult Population, n = 563^a^
C_max, ss_, μg/mL	306 (22.1) [267, 350], 312 (63)	330 (25.0) [306, 355], 340 (88)	325 (24.5) [305, 347], 335 (84)	311 (20.3) [306, 316], 317 (66)
C_min, ss_, μg/mL	42 (61.7) [30, 60], 48 (25)	54 (55.3) [47, 63], 61 (30)	52 (57.0) [45, 60], 59 (30)	46 (57.2) [44, 48], 53 (30)
C_avg, ss_; μg/mL	92 (42.9) [71, 118], 99 (37)	116 (32.5) [106, 127], 122 (38)	111 (35.5) [101, 122], 117 (39)	100 (34.6) [98, 103], 106 (39)
AUC_ss_, day μg/mL	2569 (42.9) [1992, 3314], 2760 (1031)	3247 (32.5) [2954, 3570], 3408 (1078)	3107 (35.5) [2832, 3409], 3285 (1090)	2811 (34.6) [2734, 2890], 2979 (1087)
V_ss_, mL	2542 (47.7) [1921, 3365], 2788 (1301)	3798 (35.7) [3425, 4212], 4008 (1263)	3521 (41.3) [3164, 3918], 3778 (1347)	5216 (12.6) [5163, 5271], 5257 (652)
V_z_, mL	2754 (51.7) [2037, 3724], 3059 (1477)	4015 (38.1) [3597, 4482], 4269 (1447)	3739 (43.4) [3344, 4182], 4041 (1515)	6037 (13.5) [5970, 6104], 6090 (806)
CL, mL/day	119 (33.1) [97, 145], 125 (40)	169 (39.4) [151, 190], 182 (72)	158 (40.8) [143, 176], 171 (71)	232 (33.1) [226, 239], 244 (80)
T_1/2α_, day	0.73 (34.4) [0.6, 0.9], 0.77 (0.25)	0.81 (32.0) [0.74, 0.89], 0.85 (0.25)	0.79 (32.4) [0.73, 0.86], 0.83 (0.25)	1.68 (13.0) [1.66, 1.70], 1.69 (0.22)
T_1/2β_, day	16.1 (30.2) [13.4, 19.3], 16.8 (5.6)	16.4 (37.4) [14.7, 18.3], 17.5 (6.8)	16.4 (35.8) [14.9, 18], 17.4 (6.5)	18.0 (27.3) [17.6, 18.4], 18.7 (5.1)

AUC, area under serum drug concentration‐time curve; C_avg_, average serum drug concentration; CI, confidence interval; CL, clearance; C_min_, minimum serum drug concentration; C_max_, maximum serum drug concentration; SS, steady state; T_1/2α_, elimination half‐life for first phase; T_1/2β_, elimination half‐life for second (terminal) phase; V_ss_, steady‐state volume of distribution; V_z_, terminal‐phase volume of distribution.

All data are presented as geometric mean (coefficient of variation [%]) [95%CI], arithmetic mean (standard deviation).

^a^ The adult population was derived from a phase 1 study, a phase 2 study, and 2 phase 3 studies (BLISS‐52, BLISS‐76) of belimumab in patients with SLE, as described by Struemper et al.^12^

### Exposure‐Response Analysis

**Figure 4:**
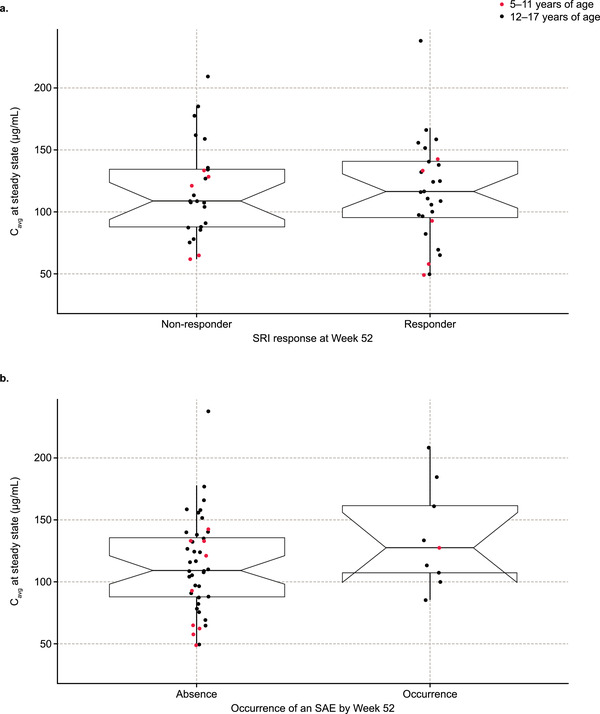
Belimumab steady‐state concentrations shown by (a) SRI4 responders and (b) SAE occurrence. C_avg_, average serum drug concentration; SAE, serious adverse event; SRI4, Systemic Lupus Erythematosus Responder Index 4. Box plots show the median (central horizontal line), interquartile range (the box, represented by the upper and lower horizontal lines), and the nearest data point no more than 1.5 times above or below the box (the whiskers, represented by the extended vertical lines). The notches, shown by the diagonal lines connecting the median to the side of the box, represent the precision in the estimate of the median value. Individual data points are superimposed for the 5‐ to 11‐year group (red points) and the 12‐ to 17‐year group (black points).

An exploratory PK/pharmacodynamic analysis on the observed data confirmed the absence of an exposure‐response relationship for efficacy and safety. The median belimumab exposure of SRI4 responders was similar to that of nonresponders, and the distribution of exposures in both groups overlapped (Figure 4A). In addition, the SRI4 response rates were similar between the 2 age groups: 50.0% (5 of 10) and 53.5% (23 of 43) for patients aged 5‐11 and 12‐17 years, respectively.

Patients experiencing or not experiencing an SAE during the 52‐week treatment period had similar median belimumab exposures, and the distribution of exposures in both groups overlapped (Figure 4B). When assessed against age, 30.0% of patients (3 of 10) and 37.2% of patients (16 of 43) in the 5‐ to 11‐year and 12‐ to 17‐year age groups, respectively, experienced at least 1 related adverse event. There was also a slightly higher rate of SAEs in the 12‐ to 17‐year age group (18.6%; 8 of 43) than in the 5‐ to 11‐year age group (10.0%; 1 of 10); however, the low patient numbers limit the generalizability of the results.

### Effects of Body Size

A post hoc analysis investigated which measure of body size (FFM, body weight, or body weight and BMI) best aligned with allometric theory. Two variants of the final popPK model were created: (1) variant 1, which is the final popPK model with the FFM covariate replaced by body weight; and (2) variant 2, which is the final popPK model with the FFM covariate replaced by body weight and BMI. Of these 3 models (the final model, variant 1, and variant 2), the final model (containing the FFM covariate) produced the body‐size exponent values on the CL and V parameters most similar to those predicted by allometric theory (0.75 for CL, 1.0 for V^20^; Table 4).

**Table 4:** Effect of Three Body Size Covariates on the Objective Function of the Final popPK Model

Body Size Covariate	Exponent Value; CL, Q	Exponent Value; V1, V2	Model Objective Function Value
FFM (final model)	0.691	0.944	5148.9
Body weight (variant 1)	0.591	0.802	5150.1
Body weight and BMI (variant 2)	0.950	1.14	5145.9
	−0.727	−0.669	

BMI, body mass index; CL, clearance; FFM, fat‐free mass; popPK, population pharmacokinetics; Q, intercompartment clearance; V1, central volume of distribution; V2, peripheral volume of distribution.

## Discussion

PLUTO is a phase 2 randomized, double‐blind, placebo‐controlled trial of belimumab 10 mg/kg intravenously in pediatric patients aged 5‐17 years with cSLE. It is the first trial to establish the PK, efficacy, and safety of belimumab in pediatric patients with cSLE, for whom belimumab has recently been approved.^15^ Previous analysis summarized the observed pre‐ and postdose belimumab concentrations,^15^ whereas the current analysis used data from 53 pediatric patients who were enrolled in the trial to provide a more detailed characterization of the PK and the exposure‐response relationships in a pediatric patient population.

Pediatric PK was found to follow 2‐compartment kinetics consistent with the popPK analysis in an adult SLE population^12^ and predicted similar steady‐state exposures for a belimumab 10 mg/kg intravenous dose. Specifically, the C_avg_ for pediatric patients (111 µg/mL) in PLUTO was close to the value observed in adult patients (100 µg/mL) for which efficacy has been established. At these pediatric and adult steady‐state exposures, there is a large molar excess of the belimumab compared with BLyS in circulation and binding to BLyS is expected to be saturated. This is supported by the absence of any efficacy and safety exposure‐response relationship in pediatric patients (Figure 4) and suggests BLyS activity is effectively neutralized at the 10 mg/kg dose. It is also consistent with the subgroup analysis with respect to pediatric age group. Younger patients aged 5‐11 years had a reduced C_avg_ at steady state compared with those aged 12‐17 years (92 vs 116 µg/mL, respectively), which was to be expected because of the allometric effects of body size with weight‐proportional dosing. However, patients aged 5‐11 years had similar SRI4 response rates to those aged 12‐17 years, meaning that the higher exposures in the 12‐ to 17‐year age group do not correspond to increased efficacy. The safety profile of belimumab in patients aged 5‐11 years was also similar to that of patients aged 12‐17 years, although the incidences of adverse events and SAEs were numerically slightly higher (by <10%) in the older patient group. This small observed difference is likely a consequence of the low numbers of patients recruited and not considered to be due to the approximately 20% higher exposure in the 12‐ to 17‐year age group. The similar belimumab exposures achieved in pediatric patients and adults and the absence of an exposure‐response demonstrated for efficacy and safety indicate that the weight‐proportional dose of 10 mg/kg approved for adults is also appropriate for pediatric patients with cSLE without any further adjustment. This is a key outcome from the current analysis.

Further comparison of the pediatric and adult PK predicts a slightly lower terminal half‐life in the pediatric population (16.3 days) compared with adults (19 days in intravenous popPK^12^ and 18 days in subcutaneous popPK^13^), which is also qualitatively consistent with allometric theory that predicts a scaling of time constants with an exponent of 0.25. Regarding the distributional phase half‐life, the geometric mean (95%CI) T_1/2α_ in adult patients was 1.68 days (1.66‐1.70 days), compared with 0.79 days (0.73‐0.86 days) for pediatric patients; this discrepancy may have reflected less frequent PK sampling in the distribution phase for the pediatric study, which could have affected the ability to correctly identify the distribution kinetics in the pediatric patients, compared with the much larger adult data set in which PK sampling in some patients was relatively frequent.

With regard to covariate selection for the full model reduction, this analysis pioneered an objective criterion for PK relevance of continuous covariates. This extended the model reduction procedure described as part of the full model approach for categorical covariates^10^ to continuous covariates, albeit in a slightly more restrictive form. Here, a covariate with questionable pharmacokinetic relevance (ie, only CIs, but not point estimates, stretch beyond the 0.8‐1.25 range) was retained in the model if the covariate parameter 95%CI did not include zero. Such an extension of the standard full model approach is worth considering for future analyses in which no meaningful a priori clinical categories exist for the tested covariates (eg, as for IgG in this analysis) or in which a continuous covariate implementation is desirable to explore and capture a unique functional relationship.

Overall, PK covariate relationships identified in the adult SLE population have been confirmed in the cSLE population of this analysis. It is of note that in this context, disease‐related covariates such as baseline IgG, proteinuria, or BLyS levels in the cSLE population of PLUTO were comparable to the corresponding values in adult SLE studies.^12^, ^13^ This was unexpected, as disease in the overall cSLE population has been shown to be more severe than in adult SLE.^21^ It is likely that the PLUTO eligibility criteria, which were modeled after the adult phase 3 trials, accounted for this similarity in terms of disease‐related baseline parameters.

Both baseline IgG and proteinuria levels were found to increase belimumab CL; such relationships are rarely investigated or identified in other monoclonal antibody population PK analyses.^22^ The IgG‐related increase in CL is mediated by increased competition for the saturating capacity of the FcRn‐receptor, which protects endogenous and exogenous IgG from endosomal lysis. This mechanism manifests itself especially in populations with hypergammaglobulinemia and has been consistently demonstrated in autoimmune diseases such as SLE^12^, ^13^ or hematological malignancies such as multiple myeloma,^23^, ^24^, ^25^ in which markedly increased IgG can occur. The proteinuria‐related increase in CL is related to the underlying disruption of the normal function of the glomerular membrane. Although intact glomeruli only filter out proteins and peptides considerably smaller than monoclonal antibodies, the disruption of the glomerular membrane through immune complex activity in SLE allows renal elimination of proteins as large as albumin or IgG, including belimumab.

The effect of body size on the CL and distribution parameters was best described in terms of FFM, which aligned more naturally to allometric theory when compared with body weight alone or body weight and BMI. When body size was described by body weight alone, the allometric exponents on CL/Q and V1/V2 were estimated at 0.591 and 0.802, respectively (variant 1; Table 4), which is below their corresponding theoretical values of 0.75 and 1.0 from allometry.^20^ A similar result was also observed in adults, with the body weight exponent on CL estimated to be 0.506.^12^ Body weight exponents on CL and V have also been estimated to be below 0.75 and 1.0, respectively, for several other monoclonal antibody therapies^26^ and not just for belimumab. It appears that for monoclonal antibodies, the theoretically expected exponents of 0.75 and 1.0 for CL and volume parameters, respectively, may apply more readily to FFM than total body weight.

This finding is supported by a previous study, in which antibody concentrations in adipose tissue were typically lower than those of several other tissues,^27^ therefore implying that the fat‐free component of body mass predominates in determining monoclonal antibody disposition and that the adipose component of total body weight is essentially inert in relation to monoclonal antibody distribution and turnover. Furthermore, including BMI as a covariate of CL/Q and V1/V2 in addition to body weight in the popPK model for belimumab acted to subtract the fat‐mass component of body weight (variant 2; Table 4). However, this crude overcorrection resulted in CL/Q and V1/V2 body weight exponents increasing beyond their expected allometric values to 0.95 and 1.14, respectively. These results imply that antibody PK may largely be determined by the fat‐free component of total body weight and that body‐size scaling of PK parameters may be more predictive when based on FFM than total body weight. This is particularly important when exposures are extrapolated to a wider pediatric population, in which body weight differences between lean children and adult‐sized teenagers can typically be much larger than the differences in their FFM.

Other covariate relationships such as age, sex, SLE disease activity, liver function, and concomitant medication use had no further impact on belimumab exposure in pediatric patients. The covariate relationships included in the pediatric model of belimumab PK (FFM, estimated glomerular filtration rate [eGFR], IgG level, proteinuria, and white blood cell count) contributed to a reduction in the unexplained variability of the popPK model and will be helpful in simulating exposure for other pediatric populations. No dose adjustments based on these covariates were indicated, given the comparable pediatric and adult belimumab exposures and the absence of an exposure‐response at the 10 mg/kg dose level.

## Conclusions

Age, sex, disease activity, and concomitant medication had no impact on belimumab exposure in pediatric patients. This indicates that no dose adjustments are required to account for patient characteristics, other than body weight for the 10 mg/kg intravenous dose. The consistency of the pediatric and adult PK data provides evidence that the belimumab 10 mg/kg intravenous dose is appropriate for patients with cSLE aged 5‐17 years, supporting the recent approval of this dose in the United States, European Union, and Japan for pediatric patients with cSLE.

## Conflicts of Interest

Richard Dimelow, Beulah Ji, and Herbert Struemper are employees of GSK and hold stocks and shares in the company.

## Funding

This study (ClinicalTrials.gov identifier NCT01649765; GSK study BEL114055) was funded by GlaxoSmithKline (GSK).

## Data‐Sharing Statement

Anonymized individual participant data and study documents can be requested for further research from www.clinicalstudydatarequest.com.

## Medical Writing Support

Medical writing support was provided by Liam Campbell, PhD, of Fishawack Indicia Ltd, UK, and was funded by GSK. Programming support was provided by Aslan Bucukoglu (a third‐party contractor; not a GSK employee) and Warren Carin of GSK.

## Supporting information

Supporting Information
